# Alagille syndrome due to a *de novo NOTCH*2 mutation presenting as prenatal oligohydramnios and congenital bilateral renal hypodysplasia: A case report

**DOI:** 10.3389/fped.2022.1020536

**Published:** 2022-11-15

**Authors:** Fengdan Xu, Qi Peng, Xiaoguang He, Xiaolan Chen, Shuanglan Jiang, Xiaomei Lu, Ning Li

**Affiliations:** ^1^ Department of Neonatology, Guangdong Medical University Affiliated Dongguan Children’s Hospital, Dongguan, China; ^2^ Department of Medical and Molecular Genetics, Dongguan Institute of Pediatrics, Dongguan, China; ^3^ Department of Ultrasonography, Guangdong Medical University Affiliated Dongguan Children’s Hospital, Dongguan, China

**Keywords:** Alagille syndrome, *NOTCH2* gene, prenatal oligohydramnios, congenitally bilateral renal hypodysplasia, case report

## Abstract

**Introduction:**

Here, we report the case of an infant suffering from Alagille syndrome (ALGS), manifesting with the atypical clinical manifestations of prenatal oligohydramnios and renal lesions. To the best of our knowledge, this is the first case of ALGS presenting as prenatal oligohydramnios and renal lesions caused by a *de novo* variant of the *NOTCH*2 gene.

**Case presentation:**

A 3-month-old male infant was hospitalized for severe malnutrition. He presented with prenatal oligohydramnios from 28^+4^ weeks of gestation. After birth, he failed to thrive and suffered from impaired motor development, thermoregulation disorders, congenital bilateral renal hypodysplasia, which initially manifested as stage 5 before improving to stage 3 chronic renal impairment, slightly elevated levels of transaminases, cholestasis, and dysmorphic facial features. We used a diagnostic screening panel of 4,047 pathogenic genes and whole exome sequencing (WES) to analyze the proband and his parents (who had normal kidneys). We found that the proband carried a *de novo* heterozygous splicing variant (c.5930-2A > G) in intron 33 of the *NOTCH*2 gene. Transcriptome sequencing confirmed that the mutation of this gene site would affect the splicing of NOTCH2 mRNA and lead to exon 33 skipping.

**Conclusions:**

Our case expands the spectrum of pathogenic variants of the *NOTCH*2 gene that are known to be associated with ALGS and characterized by prenatal oligohydramnios and renal lesions. It also reminds us of the necessity to monitor the liver and kidney function of the infant if a mother has oligohydramnios during pregnancy and we recommend ALGS as an additional differential diagnosis in prenatal renal abnormalities.

## Introduction

Alagille syndrome (ALGS, MIM# 118450) is a complex autosomal dominant multisystem disorder that was first reported by Alagille in 1969; the first diagnostic criteria were reported in 1975. The incidence of ALGS is estimated to be 1:30,000 to 1:70,000 live births (1–3). The typical clinical manifestations of ALGS include liver histology with bile duct paucity; in addition, three of five major clinical manifestations are required for a firm diagnosis: cholestasis, characteristic facial features, ophthalmological abnormalities, skeletal abnormalities, and cardiac defects. In addition to these five manifestations, abnormalities of the kidneys and vasculature are also important features of ALGS (4, 5). ALGS is caused mainly by *JAG*1 gene mutation (98%) and *NOTCH*2 gene mutation (2%) (1). The clinical manifestations of *JAG*1-related ALGS are typical; however, the manifestations of ALGS caused by *NOTCH*2 gene mutation are not typical (6), thus making it easy to misdiagnose this condition. Our case was a male baby who was diagnosed with ALGS. He carried a pathological *NOTCH*2 gene mutation and presented with prenatal oligohydramnios and congenitally bilateral renal hypodysplasia, manifested as chronic kidney disease from initial stage 5, then improved to stage 3. We also present detailed clinical data and prenatal and postpartum ultrasound data. Our case provides a good reference for the future diagnosis of *NOTCH*2-related ALGS patients.

## Case presentation

**Figure 1:**
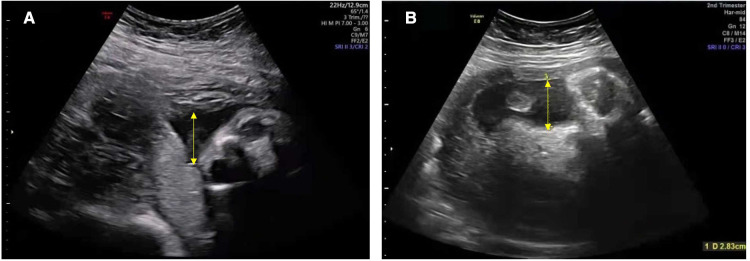
Ultrasound showing a reduced volume of amniotic fluid in the mother during pregnancy. The amniotic fluid index and the maximum depth (yellow arrow): (**A**) 81 mm and 33 mm at 28^+4^ weeks; (**B**) 47 mm and 28 mm at 34^+5^ weeks (reference ranges for the index of amniotic fluid and the maximum depth: >80 mm and >30 mm), respectively.

The proband's parents and elder brother are healthy, and his parents are not close relatives. His mother received antepartum examinations regularly during pregnancy; prenatal oligohydramnios was discovered at 28^+4^ weeks of gestational age. Three days before delivery, the amniotic fluid index and the maximum depth gradually reduced to 47 and 28 mm, respectively (Figure 1). The proband was a male infant born at 35^+1^ weeks of gestational age by Cesarean section delivery due to prenatal oligohydramnios. The Apgar score was 10 at 1, 5, and 10 min after birth and the birth weight was 2,000 g (between the 3rd and 10th percentiles), thus suggesting that the infant was small for gestational age.

Dysmorphic facial features were identified in the baby, including a characteristic triangular appearance, a broad forehead, a pointed chin, and a bulbous tip of the nose; as described in the previous literature (3). In addition, his hair and eyebrows were extremely sparse, which had not been reported in the previous literature.

Following delivery, the infant failed to thrive. He was breastfed but presented with poor sucking and weight gain. He was admitted to the local hospital at 1 month and was placed on an amino acid formula; however, this failed to have any effect on weight gain. Subsequently, he was admitted to our hospital at 3 months of age and placed on a high calorific formula; his weight gain improved rapidly to the normal range from 4 to 6 months of age. However, from around 7 months, the baby's appetite and weight gain gradually deteriorated again. His weight was 7.4 kg (8%, between the 3rd and 10th percentiles), his body length was 67.5 cm (8%, between the 3rd and 10th percentiles), and his head circumference was 43 cm (11%, 10th percentiles) at 9 months; his weight was 8.2 kg (less than the 3rd percentile) with a body length of 76 cm (less than the 3rd percentile) at 1.5 years.

The infant also presented with impaired motor development. The Bayley Scales of Infant Development II (BSID-II) Motor Scale and the Peabody Developmental Motor Scale II (PDMS-2) were administered to him at 3 months of age (corrected gestational age: 2 months), which showed a mildly low score with a Gross Motor Quotient (GMQ) of 87, a Fine Motor Quotient (FMQ) of 82, a Total Motor Quotient (TMQ) of 83, and a Psychomotor Development Index (PDI) of 84. Following physiotherapy, the situation improved. When he was 9 months old (corrected gestational age: 8 months), the infant could turn over confidently and sit steady but could not climb. He could only take a few steps on his own and pronounce the words “baba, mama” but could not say other words or sentences even when he was 1.5 years old.

Furthermore, the baby could not regulate his body temperature well and could not sweat before the age of 7 months. He presented with fever (a maximum body temperature exceeding 38°C) in the outdoor environment in summer, and his body temperature decreased to normal when he went in the shade. This phenomenon stopped after a sudden sweat one day at about 7 months, and he was able to regulate his body temperature normally thereafter.

The infant suffered from chronic renal function damage; this was first characterized by elevated serum levels of creatinine (Scr) since day 3 after birth, with an estimated glomerular filtration rate (eGFR) of 5.2 ml/min/1.73 m^2^. Gradually, the serum levels of Scr and blood urea nitrogen (BUN) exceeded standard levels (7). According to the Guidelines for Chronic Kidney Disease (CKD) in Children (8), he suffered from stage 5 CKD at birth, which then improved to stage 3 from 9 months (with eGFR 34.2 ml/min/1.73 m^2^) to 1.5 years (with eGFR 36.5 ml/min/1.73 m^2^). His liver function tests were abnormal, including increased total bilirubin (TBIL, 131.2 µmol/L; reference range: 5.4–22 µmol/L), direct bilirubin (DBIL, 22.2 µmol/L; reference range: 0.4–6.2 µmol/L), glutamyl transpeptidase (GGT, 598 U/L; reference range: 9–150 U/L), and alkaline phosphatase (ALP, 324 U/L; reference range: 20–281 U/L) on day 3 after birth. Elevated levels of alanine transaminase (ALT, 61 U/L; reference range: 0–41 U/L), aspartate aminotransferase (AST, 86 U/L; reference range: 0–38 U/L), total bile acid (TBA, 109 µmol/L; reference range: 0–12 µmol/L), and phosphorus (P, 3.22 mmol/L; reference range: 1.6–3.1 mmol/L) were evident when he was 1 month old. Elevated levels of triglycerides (TG, 2.21 mmol/L; reference range: 0.7–1.7 mmol/L) were evident when he was 3 months old. Elevated levels of cholesterol (CHoL, 8.88 mmol/L; reference range: ≤5.17 mmol/L) were also evident at 6 months of age. The levels of TBIL and DBIL returned to normal 2 months after birth; however, other indicators continued to be abnormal. There was no metabolic acidosis. Additional testing included blood routine tests, TORCH, viral hepatitis tests, autoantibody tests, and plasma amino acids, and the urine organic acid profile was normal (Table 1).

**Table 1:** Biochemical test results for the patient.

Items/age	3D	1M10D	1M17D	1M19D	Items/age	3M6D	5M2D	6M11D	6M22D	7M29D	9M2D	12M	16M
ALT (U/L,0–41)	4	**61**	**70**.**1**	**77**.**1**	ALT (U/L,8–71)	52	**100**	**102**	**142**	**126**	**127**	51	**102**
AST (U/L,0–38)	17	**86**	**78**.**5**	**58**.**8**	AST (U/L,21–80)	80	68	70	**88**	**98**	**132**	64	**86**
GGT (U/L,9–150)	**598**	**392**	**798**.**1**	**1850**	GGT (U/L,6–31)	**312**	**663**	**478**	**659**	**558**	**425**	**331**	**514**
ALP (U/L,20–281)	**324**	**525**	**695**.**7**	249.4	ALP (U/L,106–420)	**496**	**759**	**806**	**652**	**915**	**892**	**615**	**634**
TBIL (µmol/L,5.4–22)	**131**.**2**	**44.6**	21.97	9.95	TBIL (µmol/L,<23)	5.6	2.7	2.8	6.3	5.6	6.6	7.7	5.1
DBIL (µmol/L,0.4–6.2)	**22**.**2**	**41.9**	**18**.**61**	**7**.**54**	DBIL (µmol/L,<4)	**4**.**1**	1.1	1.5	3.1	1.6	1.8	1.5	1.0
TBA (µmol/L,0–12)	7	**109**	**80**.**7**		TBA (µmol/L,0–15)	13.4	**25.6**	**48**.**9**	**27**.**3**	**31.5**	**37**.**7**	**69**.**8**	**30**.**4**
ALB (g/L,38–54)	34.8	44.1	46.8	38.9	ALB (g/L,39–54)	**35**.**9**	43.1	40.4	41.5	44.9	43.9	43.4	47.9
BUN (mmol/L,1.43–6.78)	6.53	**34.35**	**30**.**47**	**19**.**34**	BUN (mmol/L,1.1–5.9)	**14**.**52**	**19.58**	**13**.**26**	**12**.**48**	**14.44**	**14**.**98**	**15**.**65**	**22**.**41**
Scr (µmol/L,27–65)	**297**.**4**	**153**	**169**.**1**	**121**.**1**	Scr (µmol/L,13–33)	**92**	**87**	**104**	**97**	**76**	**72**	**91**	**76**
Height (cm)	42	51			Height (cm)	56	62				67.5		76
eGFR[ (ml/[min.1.73 m^2^)]^a^	**5**.**2**	**12.2**			eGFR (ml/[min.1.73 m^2^)]	**22**.**2**	**26.0**	** **	** **	** **	**34**.**2**	** **	**36**.**5**
K (mmol/L,3.5–5.5)	4.9	5.05	4.26	4.24	K (mmol/L,4.2–5.9)	**4**.**1**		5.2	4.7		4.6	4.4	
Na (mmol/L,136–145)	137	**130**	**131**.**9**	**134**.**4**	Na (mmol/L,134–143)	138		137	**133**		134	137	
Ca (mmol/L,2.03–2.54)	2.24	2.47	**2**.**57**	**2**.**59**	Ca (mmol/L,2.1–2.8)	2.45		2.42	2.44		2.51	2.41	
Mg (mmol/L,0.7–1.1)	0.96	**1.57**	**1**.**6**	**1**.**44**	Mg (mmol/L,0.75–1.02)	**1**.**24**	** **	** **	**1**.**52**	** **	**1**.**4**	**1**.**12**	** **
P (mmol/L,1.6–3.1)	1.99	**3.22**	**3**.**14**	2.64	P (mmol/L,1.48–2.2)	**2**.**81**	** **	** **	**2**.**81**	** **	**3**.**26**	**2**.**32**	** **
					HCO_3_^−^ (mmol/L,22–29)		24.5	**15**.**3**	**10**.**3**	25.9	**18**.**8**	23.5	21.7
					Lac (mmol/L,0.5–2.2)						**3**.**02**	** **	** **
					CHoL (mmol/L,≤5.17)	4.77		**8**.**88**	**6**.**67**		**7**.**11**	** **	**8**.**0**
					TG (mmol/L,0.7–1.7)	**2**.**21**		**9**.**61**	**3**.**93**		**6**.**77**	** **	**2**.**37**
					LDL-C (mmol/L,2.07–3.1)	**1**.**84**		2.83	**4**.**04**		2.31		2.90

Abnormal values are presented in bold. ALT, alanine transaminase; AST, aspartate aminotransferase; GGT, glutamyl transpeptidase; ALP, alkaline phosphatase; TBIL, total bilirubin; DBIL, direct bilirubin; TBA, total bile acid; ALB, albumin; BUN, blood urea nitrogen; Scr, serum creatinine; eGFR, estimated glomerular filtration rate; K, potassium; Na, sodium; Ca, calcium; Mg, magnesium; P, phosphorus; HCO_3_^−^, bicarbonate; Lac, lactic acid; CHoL, cholesterol; TG, triglycerides; LDL-C, low-density lipoprotein.

Classification of the stages of chronic kidney disease (CKD) (8): Stage 1: GFR ≥ 90 ml/min/1.73 m^2^; Stage 2: GFR: 60–89 ml/min/1.73 m^2^; Stage 3: GFR: 30–59 ml/min/1.73 m^2^; Stage 4: GFR: 15–29 ml/min/1.73 m^2^; Stage 5: GFR: <15 ml/min/1.73 m^2^ or dialysis.

^a^ eGFR = 0.413 × [height(cm)/Scr(mg/dl)] (7).

**Figure 2:**
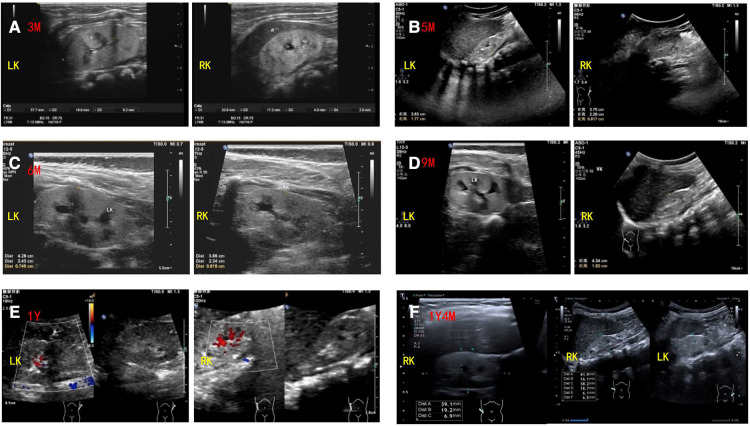
Renal ultrasound findings of the patient. Ultrasound showed bilateral hypodysplastic kidneys with enhanced echogenicity and obtained corticomedullary differentiation. The size and thickness of left and right kidneys of the patient are as follows: (**A**) 3 months: 38 mm × 19 mm, 6.2 mm and 34 mm × 17 mm, 4.8 mm (reference range: 55.7–64.1 mm × 32.2–36.2 mm, 23.1–28.1 mm and 55.4–63.8 mm × 29.4–33.4 mm, 22.3–26.9 mm); (**B**) 5 months: 38 mm × 22 mm, 8.1 mm and 36 mm × 17 mm, 6.4 mm (reference range: 55.7–64.1 mm × 32.2–36.2 mm, 23.1–28.1 mm and 55.4–63.8 mm × 29.4–33.4 mm, 22.3–26.9 mm); (**C**) 6 months: 43 mm × 24 mm, 7.4 mm and 41 mm × 21 mm, 8.0 mm (reference range: 55.7–64.1 mm × 32.2–36.2 mm, 23.1–28.1 mm and 55.4–63.8 mm × 29.4–33.4 mm, 22.3–26.9 mm); (**D**) 9 months: 45 mm × 25 mm, 7.4 mm and 43 mm × 21 mm, 7.6 mm (reference range: 62.3–67.3 mm × 34.2–37.2 mm, 24.9–29.7 mm and 61.6–67.4 mm × 33.7–37.3 mm, 23.7–30.1 mm); (**E**) 1 year: 41 mm × 19 mm, 8.1 mm and 42 mm × 18 mm, 6.8 mm (reference range: 62.3–67.3 mm × 34.2–37.2 mm, 24.9–29.7 mm and 61.6–67.4 mm × 33.7–37.3 mm, 23.7–30.1 mm); (**F**) 1 year and 4 months: 40 mm × 19 mm, 6.1 mm and 41 mm × 19 mm, 6.5 mm (reference range: 62.3–67.3 mm × 34.2–37.2 mm, 24.9–29.7 mm and 61.6–67.4 mm × 33.7–37.3 mm, 23.7–30.1 mm).

Abdominal ultrasound revealed bilateral hypodysplastic kidneys (Figure 2). The liver and biliary systems appeared normal. Cardiac ultrasound, cranial magnetic resonance imaging (MRI), spinal MRI, and eye examination (including fundus and slit lamp examination) revealed no obvious abnormalities.

**Figure 3:**
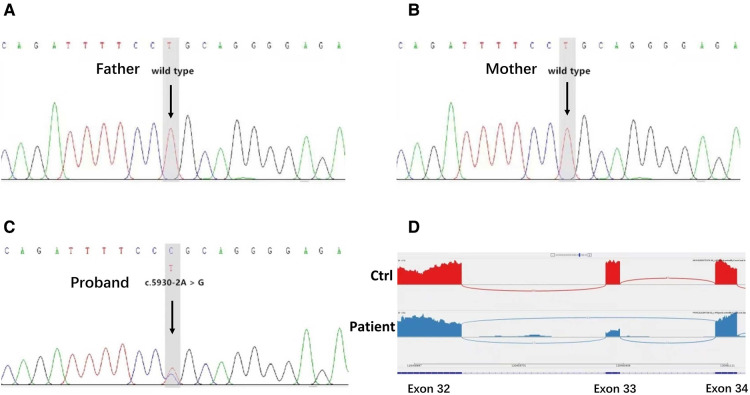
Genetic test results. (**A,B**) Sanger sequencing of the targeted *NOTCH*2 variation in the proband's family members. The arrows indicate that the patient's parents possessed the wild-type genotype for *NOTCH*2. (**C**) Identification of a *NOTCH*2 pathogenic variant in the patient. The arrows indicate an alteration from A to G in the *NOTCH*2 gene of the patient. (**D**) Transcriptome sequencing confirmed that the mutation of this gene site would affect the splicing of *NOTCH*2 mRNA and lead to exon 33 skipping.

Target genes were captured with the Clinical 4000 Pathogenic Gene Package (Guangzhou Jia-Jian Medical Testing, Guangzhou, China), which includes 4,047 genes associated with known Mendelian genetic diseases and genes for typical renal abnormalities like HNF1B and other renal ciliopathies and is based on the OMIM database (https://omim.org/). We identified a heterozygous variation in intron 33 of the *NOTCH*2 gene (c.5930-2A > G) but no variants of the *JAG*1 gene or any other variants of interest. The family members of the patient were also tested for the target variation by Sanger sequencing. His parents did not carry this mutation, suggesting that this was a *de novo* mutation. His parents had none of the clinical abnormalities seen in the baby. Transcriptomic sequencing was used to verify the splice site change at the RNA level caused by the variant, thus confirming that the c.5930-2A > G variant could lead to the skipping of exon 33, thereby leading to alterations in NOTCH2 mRNA splicing (Figure 3).

Next, we performed whole exome sequencing (WES) to analyze the coding exons and the exon–intron boundaries of protein-coding genes (Cipher Gene, Beijing, China), which included a special focus on genes affecting renal abnormalities. However, in addition to the c.5930-2A > G mutation in NOTCH2, no other genetic mutations were detected that matched the patient's phenotype.

In conclusion, ALGS was diagnosed by considering the results of molecular diagnosis, clinical features, ultrasound, and other auxiliary examinations.

## Discussion and conclusions

ALGS is a hereditary multiple-organ disease that is associated with significant variability in severity. Patients may show different patterns and degrees of organ involvement (1). Mild cases only show some subclinical manifestations, while severe cases show serious liver or heart involvement that may even be life-threatening. In the case reported herein, the patient showed various features, including prenatal oligohydramnios, failure to thrive, motor developmental impairment, and dysfunctional thermoregulation; this condition has not been reported in the previous literature. The patient also suffered from a sweating disorder for a certain period, congenital bilateral renal hypodysplastic, which manifested initially as stage 5 and then improved to stage 3 CKD, slightly elevated transaminases, cholestasis, and dysmorphic facial features. To further determine the pathogenic factors involved, genetic testing was carried out to accurately diagnose the disease. Finally, the patient was diagnosed with Alagille syndrome considering both his phenotype and genotype.

Because the clinical features of this disease are diverse and the disease lacks genotype–phenotype relationships, this creates a significant challenge for accurate diagnosis (7). At present, the diagnosis of ALGS mainly includes liver histology showing bile duct deficiency and three out of five main clinical manifestations, including cholestasis, skeletal abnormalities, congenital cardiac defects, ophthalmological abnormalities, and characteristic facial features. If the first-degree relatives of the patient had been diagnosed with ALGS, then this is considered sufficient for the patient to be diagnosed with ALGS as long as the patient met the two classic criteria (8, 9). *NOTCH*2 gene mutation was identified as a pathogenic gene in the JAG1/NOTCH2 double heterozygous mouse model in 2002 (10). In 2006, McDaniell et al. found that 5 out of 11 ALGS probands who were *JAG*1 mutation-negative possessed a *NOTCH*2 mutation and showed renal involvement, including small kidneys with cysts bilaterally and dysplastic kidneys (11). In another study, Gilbert et al. showed that liver involvement was common in NOTCH2 probands and that the prevalence of ophthalmological and renal abnormalities (manifest as renal dysplasia) was similar to that of JAG1 patients (6). However, compared with the JAG1 (+) cohort, NOTCH2 (+) probands possessed a significantly lower frequency of vertebral abnormalities and facial features and less cardiac involvement. Recently, several larger studies have also shown that ALGS patients are prone to renal and vascular abnormalities (6, 12, 13). Based on these findings, the current diagnostic criteria for ALGS were adjusted as follows: if three of the seven characteristic clinical criteria are met, then there is a sufficient case for clinical diagnosis. In the absence of a molecular diagnosis or family history, a diagnosis can be made only if at least three organs are involved (7). Liver biopsy is no longer considered a mandatory means of diagnosing ALGS as the presence of cholestasis can already meet this diagnostic criterion (12). Hence, considering the young age of our patient, we did not perform a liver biopsy. Our patient had cholestasis (increased serum bile acids, high GGT, elevated cholesterol and triglycerides, and failure to thrive) along with renal abnormalities. He also had dysmorphic facial features; however, in previous reports, this rarely occurs in individuals with *NOTCH*2 mutation. In addition to the above clinical manifestations, our case also showed prenatal oligohydramnios from 28^+4^ weeks of gestational age. This condition has not been described previously in the literature. We considered that this condition was related to renal hypodysplasia, reminding us of the necessity to monitor the liver and kidney function of the infant if their mother has oligohydramnios during pregnancy. It is interesting to note that this patient had transient abnormal thermoregulation; this has not been reported previously. First, we considered the possibility that he might suffer from ectodermal dysplasia and suggested performing a skin biopsy to examine the infant's hair follicles and sweat glands, but the parents refused this examination. We did not find any indication in the literature that ALGS may be associated with ectodermal dysplasia, and neither a screening panel of 4,000 pathogenic genes or a WES test identified any gene mutations related to ectodermal dysplasia or any other abnormal thermoregulation. Further research is required to confirm whether mutation of the *NOTCH*2 gene is involved. Close follow-up showed that the child had no other abnormal clinical manifestations except that his temperature changed with the environment; the child's thermoregulation ability returned to normal after the age of 7 months. Of course, we cannot exclude the fact that this condition was caused by the poor nutritional status and the insufficient regulatory capacity of the body of the child at that time; however, this needs to be confirmed by further research.

Although we have a deeper understanding of the clinical characteristics of ALGS, there is no effective method to treat this condition. Current therapeutic strategies for ALGS are mainly symptomatic support treatments that focus on solving the symptoms of each patient (14–17). The prognosis and risk of death depend on the severity of the liver, kidney, heart, and the other organs involved. Previous reports show that the main causes of death include severe liver diseases, such as necessitating liver transplantation (25%), intracranial hemorrhage (25%), and complex congenital heart disease (15%) (18). The 20-year life expectancy is 75% of all these patients (18). Our case did not receive any special treatment; our approach was mainly focused on guiding rational feeding, regular follow-up, monitoring growth and development, and monitoring changes in liver and kidney functions. This child easily gets sick and had been treated in the outpatient and emergency department due to upper respiratory tract infection, diarrhea, and other diseases five times in the past year. We advised the patient's family to avoid using drugs that might damage the patient’s liver and kidney function. We have continued to follow up on the case to this day.

## Conclusion

Our case expands the spectrum of pathogenic variants of the *NOTCH*2 gene that are known to be associated with ALGS and characterized by prenatal oligohydramnios and renal lesions. It reminds us of the necessity to monitor the liver and kidney function of the infant if their mother has oligohydramnios during pregnancy, and we recommend ALGS as an additional differential diagnosis in prenatal renal abnormalities.

## Data Availability

The datasets presented in this study can be found in online repositories. The link is: https://www.ncbi.nlm.nih.gov/clinvar/variation/1703257, the accession number is: SCV002568359.
